# The clinically relevant MEK inhibitor mirdametinib combined with D-cycloserine and prediction error disrupts fear memory in PTSD models

**DOI:** 10.1038/s41398-024-03190-6

**Published:** 2024-12-18

**Authors:** Sanket B. Raut, Fanny Joly, Nikolas K. Haass, Rajaraman Eri, Juan J. Canales, David M. Benedek, Robert J. Ursano, Luke R. Johnson

**Affiliations:** 1 School of Psychological Sciences, College of Health and Medicine, University of Tasmania, Tasmania, TAS Australia; 2 INSERM U1216, Grenoble Institute of Neurosciences (GIN), University of Grenoble—Alpes (UGA), Grenoble, France; 3 Frazer Institute, The University of Queensland, Woolloongabba, QLD Australia; 4 School of Science, STEM College, RMIT University, Melbourne, VIC Australia; 5 School of Psychology, Victoria University, Wellington, New Zealand; 6 Center for the Study of Traumatic Stress, Department of Psychiatry, Uniformed Services University School of Medicine, Bethesda, MD USA; 7 School of Medicine, University of Queensland, Brisbane, QLD Australia

**Keywords:** Psychiatric disorders, Pharmacology, Learning and memory

## Abstract

This study establishes mirdametinib as the first MEK inhibitor that can undergo clinical development for psychiatric indications such as post-traumatic stress disorder (PTSD). PTSD is characterized by persistent traumatic memories with limited effective treatment options. A body of evidence suggests that memory storage is dynamic and constantly updated through post-retrieval modification a process termed reconsolidation. Although ERK/MAPK signaling plays a central role in fear memory consolidation, no clinically translatable MEK inhibitor has been tested in experimental models or in clinical trials to disrupt this process. Furthermore, there is need to develop pharmacological and behavioral strategies to labilize the memory to make it susceptible for disruption. Here, we disrupted fear memory reconsolidation with the clinically relevant MEK inhibitor mirdametinib in C57BL/6 mice and tested memory destabilization strategies using an auditory fear conditioning paradigm, with drugs administered following reactivation of memory. We found prediction error effective in labilizing weak fear memory and combined D-cycloserine (DCS) and predication error effective in labilizing strong fear memory. Mirdametinib disrupted the weak fear memory and reduced ERK phosphorylation in lateral amygdala when coupled with prediction error at the time of memory reactivation but required coordinated combination of DCS, prediction error and mirdametinib to disrupt strong fear memory. Barnes maze spatial memory test and open field test revealed that mirdametinib did not affect retrieval of other forms (spatial) of long-term memory and locomotor activity. Furthermore, the effect of mirdametinib was specific to reconsolidation as it had no effect on fear memory when given without reactivation. These translational findings identify a new drug that can be adapted for the treatment of PTSD.

## Introduction

Post-traumatic stress disorder (PTSD) develops following exposure to trauma and is associated with significant comorbidities and reduction in quality of life [1–4]. A key pathological hallmark of PTSD is dysfunction of fear memory which results in symptoms such as flashbacks, nightmare and avoidance of places, activities or people that remind the person of trauma [5, 6]. Data from WHO World Mental Health Surveys indicate that lifetime prevalence of PTSD across 24 surveyed countries is 3.9% in the general population and 5.6% among the trauma exposed individuals [7]. Trauma-focused psychotherapies are effective in treating PTSD, but they have significant drawbacks, such as long treatment times and a lack of therapist availability and low uptake [8]. Selective serotonin reuptake inhibitors (SSRIs) remain the established pharmacological treatment for PTSD and while they are more effective than placebo, they only have a small effect and 43% of patients who take them do not fully respond [9, 10]. Furthermore, while the current pharmacological approaches including SSRIs and serotonin and norepinephrine reuptake inhibitors (SNRIs) aim to improve patients’ mood [11, 12] there is lack of approved drugs which target fear memory to provide long term solution for PTSD patients.

Pharmacological modification of a post-retrieval trauma-associated fear memory has emerged as potential innovative treatment strategy for PTSD. Reactivation of the fear memory trace through recall triggers lability, requiring re-storage through protein synthesis-dependent reconsolidation processes [13–15]. Post-retrieval modification of memory is necessary to incorporate new information to update the memory; however, during the period of lability window, memories are susceptible to modification by pharmacological and behavioral interventions [16–19]. Disruption of traumatic memories may offer a long-term solution for PTSD as maladaptive memories contribute to treatment resistance [20]. Pharmacological disruption of traumatic fear memories is increasingly being explored as a unique approach to offer a long-term solution to patients with PTSD [21–23]. In clinical trials on memory reconsolidation therapy in PTSD patients, propranolol has been the most studied drug. Results from these clinical trials show that while propranolol was effective in reducing symptoms of PTSD in some studies, it failed to show consistent improvement in PTSD symptoms in later clinical trials [20, 23, 24]. However, our recent meta-analysis showed that propranolol, the most commonly studied drug to disrupt fear memory, did not significantly reduce PTSD symptoms and physiological responding, except for heart rate in patients with PTSD [25]. Moreover, drugs targeting the mTOR pathway (sirolimus) [26] and glucocorticoid receptors (mifepristone) [23] also failed to disrupt fear memories in PTSD patients. Therefore, there is need to test clinically translatable drugs and approaches that can be adapted for treatment of PTSD.

Consolidation of fear memories requires key cellular signaling cascades within discrete brain regions in the fear-learning circuit [27–29]. Notably, these include the ERK/MAPK cascade in the lateral amygdala (LA) [18, 27, 30]. Pharmacological inhibitors of MEK, the kinase that phosphorylates ERK, have been shown to disrupt both consolidation and reconsolidation of fear memories [28, 31]. However, previous research used either intracerebral administration of U0126, a MEK inhibitor with poor brain permeability, to localize mechanisms of reconsolidation [28, 29], or systemic administration of SL327, an experimental compound not used clinically due to its toxicity [31]. Furthermore, the current MEK inhibitors approved for human use for cancer treatment (e.g., trametinib, cobimetinib) have poor brain permeability [32]. As a result, MEK inhibitors have not been tested clinically for PTSD. The MEK inhibitor, mirdametinib (PD0325901), which completed Phase 2 clinical trial for neurofibromatosis type 1 patients [33], was shown to cross the blood brain barrier and block activation of ERK [34]. Moreover, intraperitoneal administration of mirdametinib impaired reward-associated memories in C57BL/6 mice [35], however its translational potential for PTSD has not yet been demonstrated.

Importantly before memories can be modified following retrieval, they must first enter an active labile and modifiable state. Memories can be made labile with both behavioral and pharmacological strategies. However, these approaches have not been extensively tested in clinically translatable models for PTSD. Evidence from preclinical and clinical studies suggests that prediction error is needed for destabilization of memory [36–40]. Furthermore, there are experimental conditions referred to as boundary conditions which restrict whether memories undergo reconsolidation [41–43]. Amongst the boundary conditions identified, memory strength is particularly important for PTSD as it is characterized by exposure to a severe traumatic event which leads to strong fear memory [16, 44]. If extremely unpleasant experiences function as boundary condition for reconsolidation, then trauma memories will be resistant to reconsolidation intervention. Initially, we tested the role of prediction error in modulating the effect of mirdametinib by changing the duration of tone [45] during memory reactivation compared to training. Then, we studied the combined effect of prediction error and N-methyl-D-aspartate (NMDA) GluN2B receptor partial agonist D-cycloserine (DCS) [46–48] on destabilization of strong fear memory. Here we combined both clinically relevant behavioral and pharmacological strategies to labilised fear memory combined with clinically relevant mirdametinib to disrupt fear memories to produce a unique and translatable approach to PTSD treatment.

## Materials and methods

### Animals

Male C57BL/6 mice weighing 25–30 g, procured from Animal Resource Centre, Western Australia, were used. Animals were maintained on a 12-h light–dark cycle (lights on from 7:30 to 19:30) at room temperature of 21°C–23°C, were tagged with ear clips for identification and tested during light period. Mice were housed 4–5 individuals per cage. Food and water were provided ad libitum throughout the experiment. Mice were acclimatized to the UTAS Newnham animal facility for 8 days. All experiments were approved by Institutional Animal Ethics Committee (AEC No.: A0018070) and were conducted according to the Australian Code for the care and use of animals for scientific purposes. Every attempt was made to ensure minimum discomfort to the animals throughout the experiments.

### Study drugs

Mirdametinib (SelleckChem S1036) was dissolved in 5% DMSO, 40% PEG 400, and 5% Tween 80 in distilled water. Three doses (5, 10, and 25 mg/kg) were selected based on the study by Papale et al. [35] which showed mirdametinib impaired reward memory and prevented induction of cocaine conditioned place preference. SL327 (SelleckChem S1066) was selected as the positive control (50 mg/kg) based on work by Cestari et al. in which SL327 was shown to attenuate fear memory by inhibiting brain MEK. SL327 was dissolved in 2% DMSO, 30% PEG 400, and 5% Tween 80 in distilled water [31]. Trametinib (SelleckChem S2673), a MEK inhibitor approved for clinical use, was used as the negative control (5 mg/kg) as it does not cross the blood brain barrier [32, 34]. Trametinib was dissolved in 4% DMSO in distilled water. D-Cycloserine (DCS, Sigma-Aldrich, Australia) was dissolved in saline (1 ml/kg) and administered intraperitoneally. The DCS (15 mg/kg) dose was selected based on the previous study where DCS facilitated the destabilization of fear memory in previously stressed animals [46]. The vehicle group received 5% DMSO, 40% PEG 400, and 5% Tween 80 in distilled water. Animals were weighed on the day of memory reactivation and study drugs were injected intraperitoneally.

### Behavioral tests

#### Fear conditioning and blockade of memory reconsolidation behavior

An auditory fear conditioning paradigm was used for experiments 1, 2, 3, 4 and 6. The experimental procedure was carried out in two different chambers (Actimetrics/Lafayette instruments, USA). Mice were trained in conditioning chamber [A] wherein a single conditioned stimulus (CS; tone 78 dB, 3 kHz for 30 s) was paired with an unconditioned stimulus (US; foot shock 0.5 mA for 2 s) using a previously reported protocol investigating the effects of the MEK inhibitor SL327 on fear memory reconsolidation [31]. Mice were returned to their home cage 30 s after the foot shock. Twenty-four hours after training, mice were re-exposed to the CS in a different chamber [B]. Chamber B had different flooring (cage bedding material), chamber dimensions and appearance, and scent (eucalyptus essence) to ensure only auditory fear memories were reactivated. Mice that did not satisfy the criteria for conditioned freezing defined as more than 37 percent freezing [49] on day 2 (memory reactivation) were removed from further experiment. Memory was assessed 24 h later (test) in context B, with the percentage of time mice spent freezing when presented with the CS used as the dependent measure. Freezing behavior (defined as complete lack of movement, except for respiration) was assessed over 3 min. Each chamber had a micro video camera at the top to record the animal behavior. Freezeframe software (Actimetrics/Lafayette instruments, USA) was used to record and analyze the freezing behavior.

#### Barnes maze test for spatial memory assessment

Barnes maze test was used to test for the effect of study drugs on spatial memory. The maze (Actimetrics/Lafayette instruments, USA) consisted of an elevated circular platform, 95 cm in diameter, containing 20 circular holes around its periphery, each 5 cm in diameter along the perimeter. A small dark recessed escape box could be attached under any of the 20 holes in the platform. Bright light provided the required aversive stimulus for animals to complete the task. Visual cues (triangle, rectangle, circle, and a cross of different colors) were placed surrounding the maze. A small tissue paper was placed in the escape box which was replaced after every trial. During habituation phase, each mouse was allowed to explore the maze for 5 min. If the animal found the escape box it was allowed to stay there for 2 min. If the animal did not find the escape box, then it was guided towards escape box and kept there for 2 min. During acquisition sessions, the location of escape box was changed from the habituation trial. The mouse was placed in the center and allowed to explore the maze for 3 min. If the mouse entered the escape box, it was allowed to stay there for 1 min. If the mouse did not find the escape box in 3 min, they were gently guided towards it and allowed to stay there for 1 min. Each mouse received 4 trails per day with inter-trial interval of 15 min on day 2, 3, and 4. On day 5, a probe trial session (90 s) was conducted similar to acquisition trials except that the escape box was removed. The following behavioral parameters were recorded: primary latency—time before the first exploration of hole with escape box; primary errors—number of errors committed before first exploration of hole with escape box; time spent in target quadrant—the maze was divided in four quadrants and the time mice spent in the target quadrant during probe trial was recorded.

#### Open field test to measure spontaneous locomotor activity

The locomotor activity was measured using a 40 × 40 cm Plexiglas box. Immediately after Barnes maze test, each mouse was placed in the box and activity was video recorded. The apparatus floor was divided by markers into 9 equal parts (3 rows of 3). Each mouse was placed individually at the center of the box and locomotor activity was recorded as number of lines crossed in 5 min.

### Procedure

#### Experiment 1

**Fig. 1:**
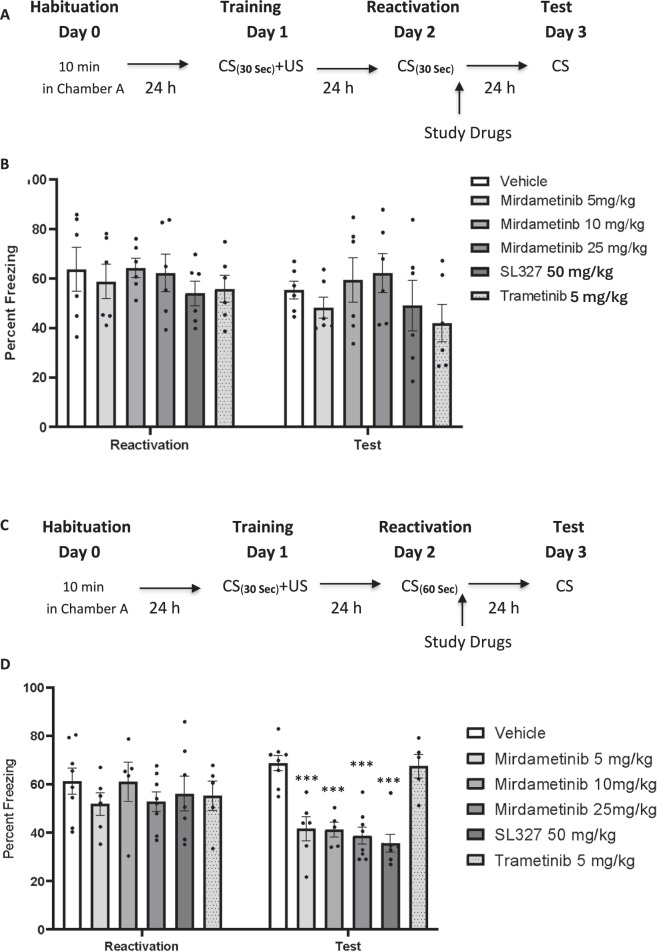
A single dose of mirdametinib (5, 10, or 25 mg/kg) given after the recall without predication error failed to disrupt fear memory but when given with prediction error at the time of memory reactivation, blocked the reconsolidation of fear memory. **A** Schematic representation of experimental protocol used. **B** Mean percentage of freezing in C57BL/6 mice with vehicle (n = 6), different doses of mirdametinib (5 mg/kg, n = 6; 10 mg/kg, n = 6; 25 mg/kg, n = 6), SL327 (50 mg/kg; n = 6) and trametinib (5 mg/kg; n = 6) in experiment 1. Data: Mean ± SE. **C** Schematic representation of experimental protocol used. **D** Mean percentage of freezing in C57BL/6 mice with vehicle (n = 8), different doses of mirdametinib (5 mg/kg, n = 6; 10 mg/kg, n = 6; 25 mg/kg, n = 8), SL327 (50 mg/kg; n = 7) and trametinib (5 mg/kg; n = 6) in experiment 2. Two-way repeated measures ANOVA. * p < 0.05, ** p < 0.01, *** p < 0.001 vs vehicle control Data: Mean ± SE.

To analyze the effect of mirdametinib on reconsolidation of auditory fear memory, mice were subjected to training, wherein a conditioned stimulus (CS) was paired with an unconditioned stimulus (US). Mice were acclimatized in chamber A for 120 s. A 30-s tone (conditioned stimulus, CS) was played (3 kHz, 78 dB). During the last 2 s of the tone, a foot shock (unconditioned stimulus, US; 0.5 mA) was administered. Both CS and US ended simultaneously, followed by a 30-s period. Mice were then returned to their home cage. After 24 h, all mice were placed in chamber B. After 120 s baseline period, a 30-s single-tone presentation reactivated the memory trace. Unlike during training, no foot shock was delivered. Immediately after retrieval, mice received injections as per following groups: Group 1: Vehicle control, Group 2–4: mirdametinib 5, 10, and 25 mg/kg, Group 5: SL327 50 mg/kg, Group 6: Trametinib 5 mg/kg. Twenty-four hours after the reactivation session, all mice were tested in chamber B. The tone was administered during the last 3 min of a 6-min test session. Freezing time was recorded and analyzed by Freezeframe software (Fig. 1A).

#### Experiment 2

To assess the role of prediction error on the effect of mirdametinib on the reconsolidation of auditory fear memory, mice were trained by pairing a CS with an US. After training, mice were randomly divided into six groups as in experiment 1. Study drugs were administered 24 h after training. Mice underwent a reactivation session on day 2. This involved re-exposing the animals to a 60 s tone CS instead of 30 s without the US, in order to induce prediction error [45]. Twenty-four hours after the reactivation session, all the groups were subjected to a test session. Freezing time was recorded and analyzed by Freezeframe software (Fig. 1C).

#### Experiment 3

**Fig. 2:**
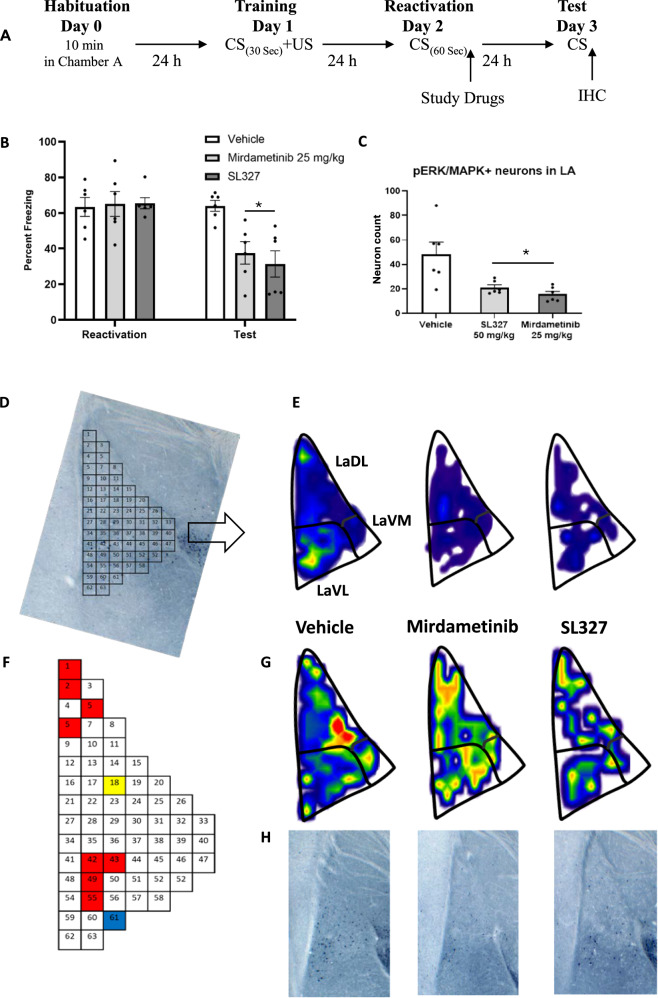
Mirdametinib high dose (25 mg/kg) and positive control SL327 (50 mg/kg), when given with prediction error at the time of memory reactivation, blocked the reconsolidation of fear memory and reduced pERK activity in LA. **A** Schematic representation of experimental protocol used. **B** Mean percentage of freezing in C57BL/6 mice with vehicle (n = 6), mirdametinib (25 mg/kg, n = 6), SL327 (50 mg/kg, n = 6) in experiment 3. Two-way repeated measures ANOVA. * p < 0.05, ** p < 0.01 vs vehicle. Data Mean ± SE. **C** Quantitative topographical analysis revealed a reduced number of pERK positive neurons in LA in mirdametinib and SL327 groups compared to vehicle control. **D** Bin matrix used for dividing the LA superimposed on pERK immunolabeled brain section. **E** Microanatomical neuron density map depicting the mean spatial distribution of activated neurons in the LA from all study groups at −1.82 Bregma. **G** Below each map is its coefficient of variance (CV) map, generated by dividing the standard deviation by the mean. **F** A visual representation of the q value matrix. q < 0.1 were depicted in color for visualization purposes. Multiple comparisons (one-way ANOVA) revealed that 10 out of 63 were statistically different (q < 0.1) between experimental conditions. Subsequently planned comparisons demonstrated that mirdametinib and SL327 reduced a comparable number of neurons (red bins) that were lower than vehicle group in 8/10 bins. In bin 61, mirdametinib was associated with lower neurons (blue bins) than vehicle and SL327 control. Bin 18 was unique to SL327, with a smaller number of pERK positive neurons (yellow bins) in comparison to vehicle and mirdametinib groups. **H** Representative pictures of each group. IHC Immunohistochemistry.

To analyze the effect of mirdametinib on ERK activity in neurons, mice were trained by pairing a CS with an US. After training, mice were randomly divided in three groups (Group 1: Vehicle control, Group 2: mirdametinib 25 mg/kg, Group 3: SL327 50 mg/kg). Memory reactivation and testing was done similar to experiment 2. On day 3 [50, 51], after the behavioral procedure mice were euthanized and brains removed for assessment of pERK levels in the lateral amygdala (Fig. 2A).

#### Experiment 4

**Fig. 3:**
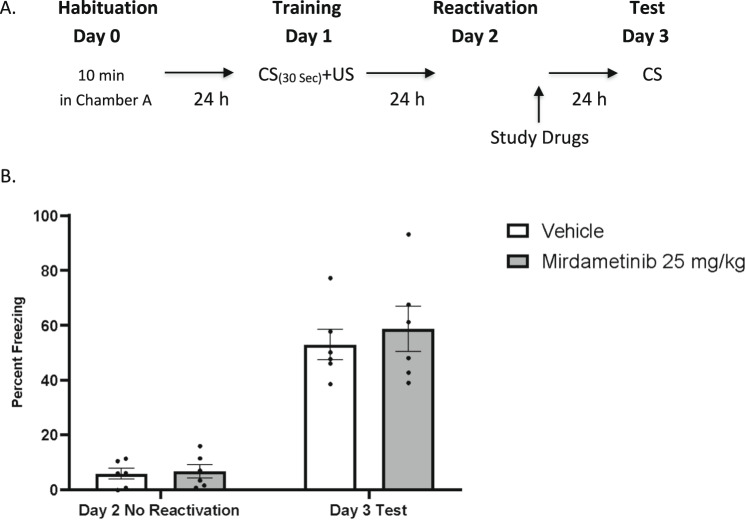
Mirdametinib failed to disrupt fear memory in absence of memory reactivation. **A** Schematic representation of experimental protocol used. **B** Mean percentage of freezing in C57BL/6 mice with vehicle (n = 6) and mirdametinib (25 mg/kg, n = 6) in experiment 4. Two-way repeated measures ANOVA. Data Mean ± SE.

To test the effect of mirdametinib specifically on memory reconsolidation, mice were subjected to training with CS and US. However, on day 2, the CS tone was not delivered to test the effect of the drug in absence of memory reactivation. Mice were randomly divided in three groups (Group 1: Vehicle control, Group 2: mirdametinib 25 mg/kg, Group 3: SL327 50 mg/kg). Study drugs were administered 24 h after the training. Memory was tested on day 3 (Fig. 3A).

#### Experiment 5

**Fig. 4:**
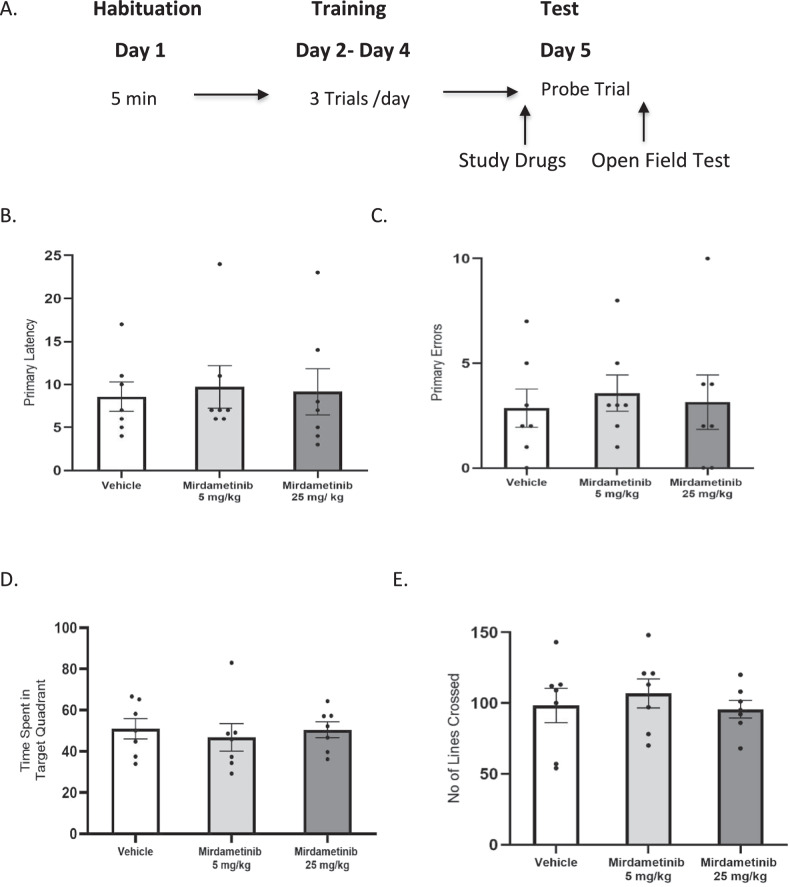
Mirdametinib does not cause memory impairment in Barnes maze test or affect locomotor activity in open field test. **A** Schematic representation of experimental protocol used. **B** Primary latency to reach escape box in Barnes maze. **C** Primary errors before reaching escape box in Barnes maze. **D** Time spent in target quadrant during probe trial in Barnes maze. **E** Number of lines crossed in open field test. n = 7/group. One-way ANOVA. Data Mean ± SE.

The effects of mirdametinib on spatial memory and spontaneous locomotor activity were tested using Barnes maze and open field test. Mice were divided into three groups (Group 1: Vehicle control, Group 2: mirdametinib 5 mg/kg, Group 3: mirdametinib 25 mg/kg). On day 1, mice were placed in maze for 5 min for habituation. From day 2 to day 4 mice were placed in the center of the maze and given 3 min time to locate the escape box. Each mouse received 4 trials/day. On day 5, 24 h after the last training day, the probe trial was conducted to test the effect of study drug on spatial memory. Study drugs were administered 1 h prior to probe trial. Immediately after probe trial, all mice underwent open field test to assess the effect of the drugs on spontaneous locomotor activity (Fig. 4A).

#### Experiment 6

Mice were subjected to training wherein conditioned stimulus (CS; tone 75 dB, 5 kHz for 30 s) was paired with an unconditioned stimulus (US; foot shock 0.6 mA for 1 s). Mice were divided into weak and strong fear conditioning groups. Mice with weak fear conditioning protocol received 1 paired tone shock presentation (1 CS-US). In strong fear conditioning group, 3 paired tone shock presentations (3 CS-US) were given over 10 min with ~1–2 min inter-trial interval [52]. Twenty-four hours after the training, 30 min before memory reactivation, mice received either DCS or saline. After reactivation session, mice were randomly divided in 8 groups (Group 1: 1 CS-US-saline-vehicle, Group 2: 1 CS-US-DCS-vehicle, Group 3: 1 CS-US-saline-mirdametinib, Group 4: 1 CS-US-DCS-mirdametinib, Group 5: 3 CS-US-saline-vehicle, Group 6: 3 CS-US-DCS-vehicle, Group 7: 3 CS-US-saline-mirdametinib, Group 8: 3 CS-US-DCS-mirdametinib). Twenty-four hours after the reactivation session all the groups were subjected to a test session and freezing time recorded for 3 min and analyzed by Freezeframe software.

### Micro-topographic mapping of pERK positive neurons in lateral amygdala

#### Tissue preparation and immunohistochemistry (IHC)

For immunohistochemistry analysis, mice from experiment 3 (vehicle, mirdametinib 25 mg/kg and SL327 50 mg/kg groups) and experiment 6 (1 CS-US-saline-vehicle, 3 CS-US-saline-vehicle, 3 CS-US-saline-mirdametinib and 3 CS-US-DCS-mirdametinib groups) were anesthetized with an intraperitoneal injection of pentobarbitone and then exsanguinated by transcardial perfusion with 20 ml 0.9% normal saline followed by 40 ml 4% paraformaldehyde (Sigma-Aldrich), 1 h after the test session. Brains were post-fixed overnight and transferred to 1X phosphate buffered saline (PBS) until processing for immunohistochemistry. Free floating sections of 70 µm were sliced with a vibratome. The sections were placed in a 1% bovine serum albumin (BSA) blocking solution for 1 h. Further, sections were incubated for 48 h at room temperature in rabbit polyclonal primary antibody to phospho-p44/42 ERK (pERK) (1:250 dilution, Cell Signaling Technology, Boston, MA). After 3 PBS washes, a secondary antibody (biotinylated goat anti-rabbit IgG, 1:200 dilution, Vector Laboratories) was added for 24 h. Sections were again washed 3 times and placed in avidin–biotin HRP complex (ABC Elite, Vector Laboratories) for 1 h. SG chromogen/hydrogen peroxide (Vector Laboratories) was used to achieve visualization of neurons with ERK activity (pERK-positive). Sections were allowed to completely dry after mounting on IHC microscope slides. Finally, sections were dehydrated in increasing percentages of alcohol (50% × 1, 70% × 1, 90% × 1, 100% × 2) followed by xylene.

#### Histology data acquisition and neuronal mapping

Histology images were acquired using an optic microscope (Olympus BX 60) at ×20 objective in a vertical mosaic manner, forming a vertical grid of 3 × 3 images for lateral amygdala (LA). Individual images where stitched using the grid/collection stitching plugin [53] in Fiji software [54] to form a single image.

Amygdala sections were anatomically matched between groups to allow for the quantitative analysis of pERK-positive neurons. Based on our previous research, the optic tract was used as consistent, identifiable landmark as it appears and lengthen when sections are placed in rostral to caudal sequence [55]. The Franklin and Paxinos mouse atlas was used to identify the position of the optic nerve [56]. The sections were matched across the groups based on the relationship between the right optic tract and the central nucleus (CeA) of the amygdala [55]. We used sections that were −1.82 mm from the bregma for this analysis. To ensure we counted consistently from the areas that include the region of interest, we traced the contours of the lateral amygdala (LA) and applied the contour to each section. The image scale was set, and the contour of LA was done manually in Fiji, using the said anatomical landmark to determine the size and edges of the target structure. The relationship between the right optic tract and the central nucleus (CeA) of the amygdala [55] is the relevant anatomical anchor to isolate the structural contour of LA (for further methods, see [55, 57]. The area of the outlined structure was measured, and pERK-positive cells present in this area were manually counted using the “multi-point” tool in Fiji. Neurons were identified by pyramidal shape of soma, primary or secondary dendrites, size between 20-40 μm and staining higher than background.

A density heat map of LA was created to visualize the special distribution of the activated neurons using the method we have previously standardized [57]. This method was followed here with slight modifications as we used Fiji software to calibrate the counting areas, draw the structures’ contours and manually count pERK-positive cells. The (x, y) coordinates of pERK-positive neurons obtained using Fiji software were binned and transformed into matrices (Origin v 9, OriginLab, Northampton, MA) to measure the exact localization and density of pERK-positive neurons. The number of bins were calculated based on existing formula [18]. Each (x, y) data were transformed into matrices containing the same number of bins to allow the comparisons between groups at the same matrix resolution. Values within each bin were assigned a color to create a final density heat map, (SigmaPlot v 12, Systat Software, San Jose. CA).

### Statistical analysis

The sample size was calculated to estimate an effect size (f) 0.58 based on the effect of the MEK inhibitor SL327 in a previous study by Cestari et.al. [31]. We used α 0.05, power 0.8, number of groups 6, number of repeated measures 3, and correlation among repeated measures of 0.5. The total sample size derived was 36 (n = 6 per group).

Results are expressed as mean ± SEM. We used two-way repeated measures ANOVA for analysis of behavioral data from reconsolidation experiments. We analyzed percentage freezing, with drug treatment as between group and test days as within group factors. Individual between-groups comparisons were performed using Tukey’s post-hoc test. We used two-way repeated measures ANOVA in this study to account for within-group variability due to repeated measures on the same animal across different test days. We used one-way ANOVA for behavioral analysis of Barnes Maze and Open Field Tests. We compared primary latency, primary errors, and time spent in target quadrant during probe trial in the Barnes maze test and number of lines crossed in the open field test.

Analysis of neuron counts was conducted using mass univariate ANOVA to assess differences in neuron activation across all conditions in each bin. False Discovery Rate (FDR) correction was conducted for multiple comparisons to minimize type II error and to identify significance in specific topographic regions of interests (ROI) [58]. FDR correction was used, with the tolerable limit set at q = 0.1. FDR correction has been applied to similar datasets previously [18, 58–60]. Comparisons were performed on the particular bin data where significant differences across conditions was found in certain micro ROIs to locate (1) the effect of the experimental versus control groups and (2) the difference between two experimental groups [59, 61]. The q-values were mapped onto the topographical matrix (bins) to reveal the highly localized topography of neuronal activation. The spatial distribution of these points of significance was confirmed on visual analysis of the neuronal topographic density maps compiled from topographic data, and also reflected earlier findings [62].

For all analyses, the alpha level of significance was set at 0.05. Statistical analyses were conducted using the Statistical Package for the Social Sciences (SPSS) v24 software (IBM Corporation, NY, USA) and GraphPad Prism v8 software (GraphPad, CA, USA).

## Results

### Mirdametinib does not disrupt fear memory reconsolidation in absence of prediction error (Experiment 1)

In the first experiment, study drugs were administered before memory reactivation; however, no prediction error was introduced during reactivation. On day 3 (test session), mice in all study groups froze similarly to the CS (tone) administration (Fig. 1B). A two-way Repeated Measures ANOVA showed no significant effect of drug (F_5,72_ = 1.56, p = 0.18) and no interaction between drug and test days (F_10,72_ = 0.91, p = 0.52), and a significant effect of test days (F_2,72_ = 143.04, p < 0.001).

### Mirdametinib combined with prediction error disrupts fear memory reconsolidation (Experiment 2)

In this experiment, we introduced a prediction error at the time of memory reactivation by administering a longer tone (60 s) (Fig. 1C) compared to experiment 1 (30 s) (Fig. 1A). Following conditioning training, some mice failed to meet the criteria for acquisition of conditioned memory when freezing was measured in the reactivation session (Day 2) and were subsequently excluded from the study. Mice were excluded as follows: 2 in mirdametinib low dose group, 2 in mirdametinib medium dose group, 2 in trametinib group and 1 in SL327 group. Therefore, the sample size for each group in experiment 2 is as follows: Vehicle: N = 8, mirdametinib low dose: N = 6, mirdametinib medium dose: N = 6, mirdametinib high dose: N = 8, SL327: N = 7 and trametinib: N = 6.

Mirdametinib was able to disrupt memory reconsolidation as indicated by reduced percentage freezing scores on day 3 (test session) (Fig. 1D). Two-way ANOVA showed a significant effect of drug (F_5,105_ = 7.40, p < 0.001), a significant interaction between drug and test days (F_10,105_ = 3.16, p = 0.001) and a significant main effect for test days (F_2,105_ = 260.57, p < 0.001). Post hoc Tukey’s test showed all three doses of mirdametinib (5 mg/kg, p < 0.001; 10 mg/kg, p < 0.001; 25 mg/kg, p < 0.001) and positive control SL327 (50 mg/kg, p < 0.001) significantly reduced freezing compared to vehicle control whereas negative control trametinib (5 mg/kg) was comparable to vehicle control (p = 0.498).

### Mirdametinib disrupts fear memory reconsolidation and reduces ERK activity in lateral amygdala neurons (experiment 3)

How mirdametinib affects the activity of neurons in LA and contributes to the disruption of memory trace is not known. The effect of mirdametinib high dose (25 mg/kg i.p.) and prediction error on fear memory reconsolidation was confirmed and their effect on ERK activity in LA neurons was studied.

Two-way ANOVA showed a significant effect of drug (F_2,45_ = 3.97, p = 0.026), a significant interaction between drug and test days (F_4,45_ = 4.06, p = 0.007) and a significant effect for test days (F_2,45_ = 124.67, p < 0.001) (Fig. 2B). Post hoc Tukey’s test showed high dose of mirdametinib (p = 0.038) and positive control SL327 (p = 0.01) significantly reduced freezing compared to vehicle control (Fig. 2B).

Further, ERK activity in LA neurons was quantified (Fig. 2C). Mirdametinib and the positive control SL327 reduced the number of neurons with ERK activity compared to vehicle (one-way ANOVA, p = 0.004) (Fig. 2C). The spatial distribution of the activated neurons was visualized by micro-topographic mapping of pERK-immunoreactive neuron density (Fig. 2D). The density maps showed reduction in neuronal activity in LaDL and LaVL subregion of LA following mirdametinib (25 mg/kg i.p.) and SL327 (50 mg/kg) administration (Fig. 2E). Multiple comparison testing showed that 10/46 bins exhibited significant differences (q < 0.1) between experimental groups (Fig. 2E). In eight of ten bins (bins 1, 2, 5, 6, 42, 43, 49, 55) the number of activated neurons in the mirdametinib and SL327 groups was significantly lower than control group (Fig. 2F). In 1/10 bins, mirdametinib group showed significantly less neurons than vehicle and SL327 groups (bin 61). Bin 18 contained a smaller number of pERK-positive neurons in the SL327 group than vehicle and mirdametinib groups. Most of the reduction in neuronal activity following mirdametinib and SL327 was limited to a relatively small fraction of the LaDL and LaVL subregion. This study provides evidence for specific effect of MEK inhibitors on spatial allocation of the auditory fear memory trace following disruption of fear memory reconsolidation.

### Mirdametinib does not disrupt fear memory reconsolidation in the absence of memory reactivation (Experiment 4)

To demonstrate that mirdametinib specifically disrupts the fear memory trace association between CS and US, we tested the effect of the highest dose of mirdametinib with the same procedure used in experiment 2 except for the reactivation of memory on day 2 (Fig. 3A). A two-way ANOVA carried out with test days as within subject and study groups as between subject factors showed no significant effect of drug (F_1,30_ = 0.52, p = 0.47) and interaction between drug and test days (F_2,30_ = 0.18, p = 0.83), but a significant effect of test days (F_2,30_ = 87.80, p < 0.001) (Fig. 3B).

### Mirdametinib does not affect spatial memory or spontaneous locomotor activity (Experiment 5)

We explored the effect of mirdametinib on spatial memory using Barnes maze test to verify that the study drug does not affect memory processes in general. Administration of mirdametinib at the low (5 mg/kg) and high dose (25 mg/kg) did not have a significant effect on primary latency (F_2,18_ = 0.06, p = 0.94) (Fig. 4B), primary errors (F_2,18_ = 0.12, p = 0.89) (Fig. 4C) and time spent in target quadrant (F_2,18_ = 0.18, p = 0.83) (Fig. 4D) compared to vehicle control. To rule out that the reduction in freezing scores in fear conditioning was due to the disruption of fear memory and not due to a general reduction in locomotor activity we performed an open field test. Result suggests that both doses of study drug did not affect locomotor activity as the number of lines crossed compared to vehicle control were not significant (F_2,18_ = 0.35, p = 0.71) (Fig. 4E).

### Pre-reactivation DCS enhanced retrieval-induced lability in memories resistant to the effect of mirdametinib on memory reconsolidation (Experiment 6)

**Fig. 5:**
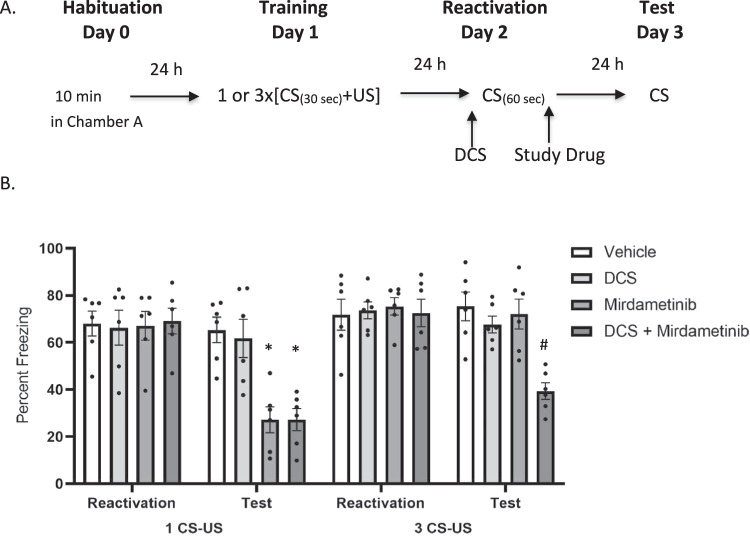
Combined administration of DCS and mirdametinib on the day of memory reactivation, blocked the reconsolidation of strong fear memory. **A** Schematic representation of experimental protocol used. **B** Mean percentage of freezing in C57BL/6 mice in 1 CS-US-saline-vehicle (n = 6), 1 CS-US-DCS-vehicle (DCS 15 mg/kg, n = 6), 1 CS-US-saline-mirdametinib (mirdametinib 25 mg/kg, n = 6), 1 CS-US-DCS-mirdametinib (DCS 15 mg/kg, mirdametinib 25 mg/kg, n = 6), 3 CS-US-saline-vehicle (n = 6), 3 CS-US-DCS-vehicle (DCS 1 mg/kg, n = 6), 3 CS-US-saline-mirdametinib (mirdametinib 25 mg/kg, n = 6), 3 CS-US-DCS-mirdametinib (DCS 15 mg/kg, mirdametinib 25 mg/kg, n = 6) groups. Two-way repeated measures ANOVA. * p < 0.05 vs 1 CS-US-saline-vehicle control. ^#^ p < 0.05 vs 3 CS-US-saline-vehicle control. Data Mean ± SE.

In this study DCS was administered before reactivation to enhance labilization followed by mirdametinib treatment. During memory re-activation (day 2), the experimental groups exhibited comparable amounts of freezing, showing that the pharmacological treatment had no effect on the retrieval process or freezing behavior. Analysis with one-way ANOVA showed no significant difference in percentage freezing between groups (p =.959) suggesting freezing was similar in both weak (1 CS-US) and strong (3 CS-US) fear memory groups (Fig. 5B).

Two-way ANOVA showed a significant effect of drug (F_7,120_ = 17.81, p < 0.001), a significant interaction between drug and test days (F_14,120_ = 2.09, p = 0.016) and a significant main effect for test days (F_2,120_ = 219, p < 0.001). Post hoc analyses showed that mice with weak fear memory (1 CS-US) administered saline or DCS before reactivation and mirdametinib after reactivation showed the expected disruption of fear memory reconsolidation as in experiment 2 (p = 0.025 and p = 0.029 respectively). Mice in the strong fear memory group (3 CS-US) that received saline before re-exposure followed by post-reactivation mirdametinib did not show reduction in freezing on test day (p = 0.891). Mice in the strong fear memory group (3 CS-US) administered DCS followed by mirdametinib showed the disruption fear memory reconsolidation as demonstrated by significant reduction in freezing on test day (P = 0.024) (Fig. 5B). These results suggest that mirdametinib inhibits the reconsolidation of strong fear memories in pre-reactivated DCS-administered animals. These findings show that DCS pre-reactivation increases destabilization of strong fear memories, thus making fear memories susceptible to disruptive effect of mirdametinib.

### Combination of pre-reactivation DCS with mirdametinib reduces ERK activity in lateral amygdala neurons after strong fear conditioning

**Fig. 6:**
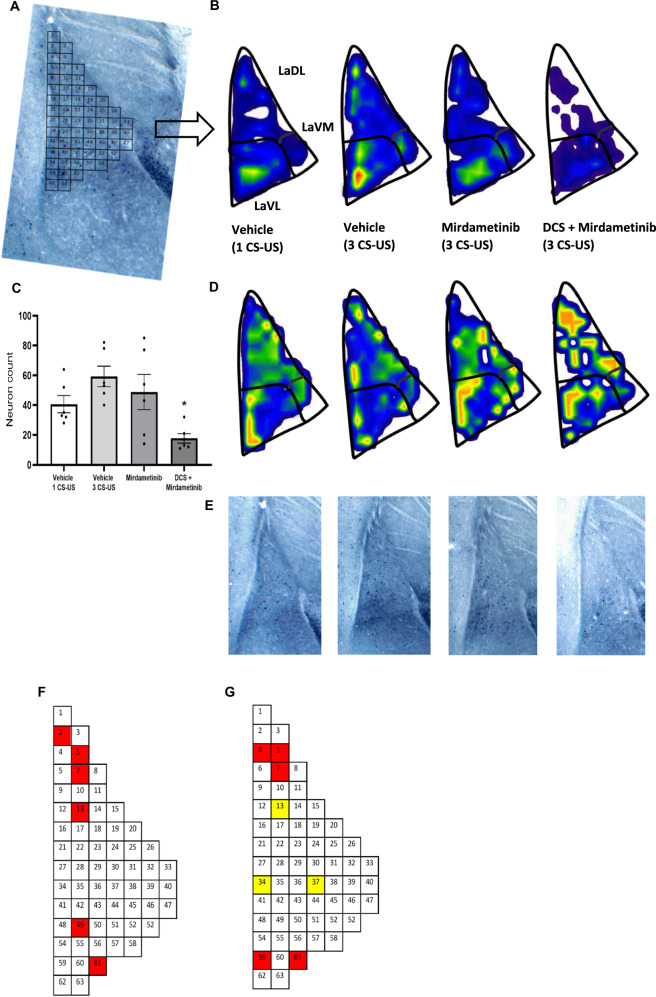
Combined administration of DCS with mirdametinib reduced pERK positive neurons in LA. **A** Bin matrix used for dividing the LA with pERK immunolabeled brain section. **B** Microanatomical neuron density map showing the mean spatial distribution of pERK expressing neurons in different parts of LA following vehicle administration in weak fear conditioning group and vehicle, mirdametinib and DCS + mirdametinib in strong fear conditioning group at −1.80 Bregma. **D** Below each map is its coefficient of variance (CV) map, generated by dividing the standard deviation by the mean. **C** Increase in number of neurons expressing pERK following strong fear conditioning (Group 1: 1 CS-US-saline-vehicle) compared to weak fear conditioning (Group 5: 3 CS-US-saline-vehicle). Combined administration of DCS with mirdametinib (Group 8: 3 CS-US-DCS-mirdametinib) reduced the number of LA neurons with pERK activity compared to vehicle (Group 5: 3 CS-US-saline-vehicle). **E** Representative pictures of each group. **F** Post hoc comparison showed increased pERK positive neurons in LaDL and LaVL following strong fear conditioning compared to weak fear conditioning. A visual representation of the q value matrix. q < 0.1 are shown in color for better visualization. Multiple comparisons testing (one way ANOVA) showed that 6 out of 63 were statistically different (q < 0.1) between weak (Group 1: 3 CS-US-sal-vehicle) and strong fear memory groups (Group 5: 3 CS-US- sal-vehicle). Quantitative topographical analysis showed significantly increased pERK immuno-positive neurons in bin 2, 5, 7, 13, 49, 61 in strong fear memory group compared to weak fear memory control. **G** Post hoc comparison showed combined administration of DCS and mirdametinib reduced pERK positive neurons in LaDL and LaVL. A visual representation of the q value matrix. q < 0.1 are shown in color for better visualization. Multiple comparisons testing (one way ANOVA) showed that 8 out of 63 were statistically different (q < 0.1) between DCS + mirdametinib group and vehicle control (3 CS-US-sal-vehicle). In three out of 63 bins (marked yellow), there was significant difference between both mirdametinib alone (Group 7: 3 CS-US-sal-mirdametinib) and DCS plus mirdametinib group (Group 8: 3 CS-US-DCS-mirdametinib) compared to vehicle control (3 CS-US-sal-vehicle). Quantitative topographical analysis showed significantly reduced pERK immuno-positive neurons in bin 4, 5, 7, 13, 34, 37, 59, 61 in DCS with mirdametinib group compared to vehicle control. LaDL dorsolateral part of LA, LaVL Ventrolateral part of LA, LaVM Ventromedial part of LA.

The ERK activity in LA neurons was quantified to compare the weak and strong fear memory trace (Fig. 6). Mice in strong fear memory group (Group 5: 3 CS-US-saline-vehicle) showed significantly higher number of neurons with ERK activity compared to weak fear memory group (Group 1: 1 CS-US-saline-vehicle) (p = 0.042) (Fig. 6A, B). Further, we studied the effect of mirdametinib alone and combined DCS and mirdametinib administration on the ERK activity in LA neurons in mice with strong fear memory (Fig. 6B). Mirdametinib alone (Group 7: 3 CS-US-saline-mirdametinib) did reduce the number pERK positive neurons but this reduction was not statistically significant (p = 0.727). The combined administration of DCS and mirdametinib (Group 8: 3 CS-US-DCS-mirdametinib) significantly reduced the number of neurons with pERK expression compared to vehicle (Group 5: 3 CS-US-saline-vehicle) (p = 0.027). We visualized the distribution of the activated neurons by micro-topographic mapping of pERK expressing immunoreactive neuron density maps (Fig. 6C).

Further, we studied the change in distribution and number of activated neurons in LA following weak and strong fear conditioning with density maps and quantitative analysis (Fig. 6D, E). Multiple comparison testing showed that 6 out of 63 bins exhibited significant differences (q < 0.1) between experimental groups (Fig. 6E). In bins 2, 5, 7, 13, 49, 61 the number of activated neurons in strong fear memory group were significantly higher than weak fear memory group (Fig. 6E). The increase in neuronal activity following strong fear conditioning was present in LaDL and LaVL subregions of LA. These data suggest that memory strength is encoded by increase recruitment of pERK positive neurons in fear memory trace in mostly the LaDL but also LaVL subregions of LA.

We also studied the change in distribution and number of activated neurons in LA following mirdametinib alone and combined DCS and mirdametinib administration in strong fear memory group with density maps and quantitative analysis. Multiple comparison testing showed that 8/63 bins exhibited significant differences (q < 0.1) between combined DCS and mirdametinib (Group 8: 3 CS-US-DCS-mirdametinib) and vehicle control group (Group 5: 3 CS-US-saline-vehicle) (Fig. 6F). In bins 4, 5, 7, 13, 34, 37, 59, 61 number of pERK positive neurons in combined DCS and mirdametinib group were significantly lower than vehicle control group (Fig. 6F). In only 3/63 bins (13, 34, 37) the number of activated neurons in the mirdametinib alone group (Group 7: 3 CS-US-saline-mirdametinib) was significantly lower than vehicle control group (Group 5: 3 CS-US-saline-vehicle) (Fig. 6F). Consistent with previous results, the reduction in neuronal activity following combined DCS and mirdametinib administration was present in LaDL and LaVL subregion of LA. These data show the effect of DCS and mirdametinib administration on spatial allocation of the strong fear memory trace following disruption of fear memory reconsolidation.

## Discussion

New and effective treatments for PTSD are urgently needed, requiring translational models and new therapeutic tools. Disruption of memory reconsolidation has offered a theoretical strategy for the development of novel treatments. However, despite considerable progress in recent decades with respect to our understanding of amygdala-based fear memory circuits, there are currently no approved treatments for PTSD based on disruption of memory reconsolidation (see Raut et al. [25]). In the present study, mirdametinib a clinically relevant MEK inhibitor, was shown to disrupt fear memory only when combined with memory destabilization strategies like prediction error and DCS at the time of memory reactivation. This effect is associated with the reduction of ERK activity in dorsolateral (LaDL) and ventrolateral (LaVL) subregions of LA. Further it was shown that mirdametinib does not disrupt other memory process like spatial memory and also has no impact on spontaneous locomotor activity at the identified doses demonstrating its functional specificity. Thus, the study strongly suggests that mirdametinib could be added to the repertoire of drugs that could be tested clinically in PTSD patients.

The vulnerability of fear memory to disruption by pharmacological intervention depends on lability of memory after reactivation. Pharmacological inhibition of memory reconsolidation processes requires memory to be destabilized into an active labile state. It is suggested that this is necessary to incorporate new information and update the memory [63]. Research suggests that prediction error at the time of memory reactivation is required for memory to enter in active labile state [36–38]. In first part of the study, all three doses of mirdametinib failed to disrupt fear memory when given after memory reactivation. Thus, failure of mirdametinib to disrupt traumatic memory could be attributed to the drug or deficiency in the experimental paradigm to reactivate the memory. To answer this, prediction error was incorporated at the time of memory reactivation in the following experiment.

Incorporation of prediction error led to disruption of fear memory by all three doses of mirdametinib. Thus, this study highlights the importance of prediction error, as mirdametinib did not affect memory in the absence of prediction error. The results in the present study are in agreement with recent memory reconsolidation literature which highlights the role of prediction error to labilize the memory [36–38]. The protein synthesis inhibitor, anisomycin, did not affect memory in the absence of prediction error while introduction of temporal prediction error at the time of retrieval led to disruption of fear memory reconsolidation [37]. Similar to the results in this study, the presentation of a CS (tone) for longer duration to introduce prediction error during reactivation led to disruption of fear memory reconsolidation by the glucocorticoid receptor antagonist mifepristone [45]. The results are in contrast to previous studies with other MEK inhibitors where memory reconsolidation was disrupted without prediction error during memory reactivation [28, 29, 64]. However, these studies involved intracerebral injection of MEK inhibitors and therefore could have achieved higher concentration compared to the systemic administration employed in this study. Researchers also used different models such as an inhibitor avoidance task [29] and an object recognition task [64] to explore the effect of MEK inhibitors on memory reconsolidation. Whether the difference in results with our study is due to the drug concentration at the site of action or variation in experimental conditions remains to be fully identified.

There is some discussion in the literature as to what constitutes prediction error and CS duration dictates susceptibility to disruption. Some authors have used negative prediction error, i.e., absence of US after presentation of CS, while others have used positive prediction error like change in CS duration and the context in which reactivation occurred, while others used change in contingency around timing of the US during reactivation [36, 37, 39].

Prediction error in the context of memory reconsolidation involves a discrepancy between what is expected and what actually occurs. A change in the conditioned stimulus (CS) duration can equate to a prediction error in the context of memory reconsolidation because it creates a discrepancy between what the subject expects based on prior conditioning and what actually occurs during the reactivation phase. During the conditioning phase, mice learn to associate a specific CS duration with an unconditioned stimulus (US). Altering the CS duration during reactivation violates these learned expectations, generating a prediction error. This aligns with the broader understanding that prediction error is fundamental to driving memory reconsolidation, as it signals a discrepancy between expected and actual outcomes, prompting the re-evaluation and updating of memory [38]. Thus, the decision to define change in CS duration as prediction error in this study was based on established terminology from previous studies [45, 46].

The difficulty in destabilization of strong memories is a critical contributing factor as to why reconsolidation therapies have not entered clinical practice. Such a boundary condition limits the circumstances under which reconsolidation of the memory will take place. None of the previous clinical studies to disrupt traumatic memories in PTSD patients used D-cycloserine to labilize memories, which might explain the lack of consistent evidence for their effectiveness [25, 65]. PTSD develops following strong aversive experience and if memory strength forms a boundary condition on reconsolidation, then pharmacological intervention to disrupt strong fear memories will fail unless the memory is adequately labilized [66, 67]. Therefore, the development of new therapeutic strategies to disrupt strong fear memories is critical as this could allow for disruption of persistent traumatic memories seen in PTSD. Mirdametinib was shown to inhibit fear memory reconsolidation in mice with weak fear memory (1 CS-US pairing), however, a strong fear conditioning protocol with 3 CS-US pairing rendered a memory trace resistant to disruption of reconsolidation by mirdametinib. Systemic administration of D-cycloserine (DCS), an NMDA receptor partial agonist, restored the disruptive effect of mirdametinib on memory reconsolidation. Our results are consistent with previous research which shows that systemic administration of DCS restored the memory disrupting effect of midazolam in mice with strong fear memory induced by prior stress [46, 47].

The ability of DCS to promote memory labilisation relies on increased NMDA receptor-mediated glutamatergic transmission [68], which is a prerequisite for the memory labilization process following retrieval [69, 70]. Consequently, the NMDA receptor activation by DCS should restore the susceptibility of strong fear memories to disruptive effect of mirdametinib. Research suggests that activity of GluN2B NMDAR subunits is crucial for inducing reconsolidation. It has been shown that activation of GluN2B is necessary for the labilization of fear memory, whereas activation of GluN2A is necessary for its subsequent restabilization [71]. The ratio of GluN2A to GluN2B subunit expression is proposed to regulate the susceptibility of memory to destablization [70]. Thus, DCS as a partial agonist of NMDA receptor may have led to destabilization of strong fear memory through activation of NMDA GluN2B receptors. Furthermore, DCS may facilitate destablization of strong fear memory by protein breakdown via ubiquitin/proteasome during memory reactivation [72, 73]. Stress prior to fear conditioning reduces the expression of polyubiquitinated proteins in the BLA after fear memory recall in rats, which is prevented by DCS administration before recall [73]. Anisomycin’s amnestic effect was blocked when proteasome inhibitor was administered prior to memory reactivation [72]. In addition, proteasome inhibitors reduced the synaptic response and fear memory extinction caused by DCS [74], demonstrating that ubiquitin/proteasome system mediated the effect of DCS on fear memory. In light of the fact that ubiquitin/proteasome system mediates memory labilization, the DCS mediated destabilization of strong fear memories may be due to the activation of this system. Further research is required to address this hypothesis.

**Fig. 7:**
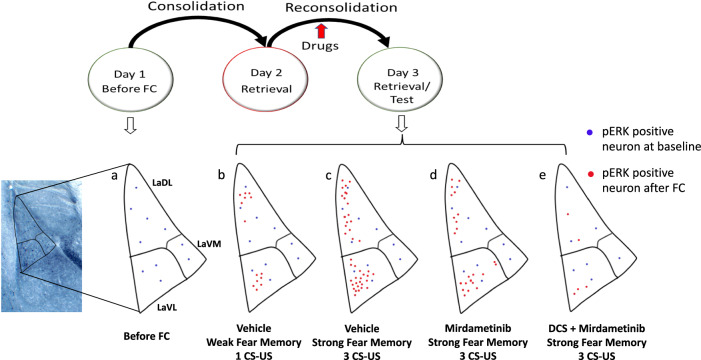
Schematic model showing distribution of pERK-positive neurons in different subregions of LA in different experimental conditions. **a** LA neurons with ERK activity at baseline before fear conditioning (FC), **b** LA neurons with ERK activity after weak fear conditioning (1 CS-US), **c** LA neurons with ERK activity after strong fear conditioning (3 CS-US), **d** LA neurons with ERK activity after mirdametinib administration in mice that underwent strong fear conditioning, **e** LA neurons with ERK activity after combined DCS and mirdametinib administration in mice that underwent strong fear conditioning.

Earlier research has shown that pERK activation in the lateral amygdala is necessary for the reconsolidation of fear memory [18, 28]. Exposure to paired CS and US significantly increase the number of pERK positive neurons in the LA compared to unpaired CS and US [27, 62] or the CS alone [27]. Earlier research in our lab has identified the spatial distribution of activated neurons (engram) in lateral amygdala following fear memory consolidation and reconsolidation [18]. Consistent with previous research, our findings demonstrated that the recall of auditory fear memory under conditions that lead to reconsolidation resulted in a clear up-regulation of pERK expression in LA. However, the impact of pharmacological modulation of fear memory on spatial distribution of activated neuron in LA has not been explored. The data from the present study has shown for the first time that dorsolateral (LaDL) and ventrolateral (LaVL) subregion of LA are critically involved in mediating the effect of drugs on fear memory trace. Quantitative mapping following fear memory reconsolidation in earlier study showed the increased number of neurons in dorsolateral region of LA [18]. The data from present study suggest that ventrolateral subregion (LaVL) plays a role in reconsolidation of the fear memory. Furthermore, quantitative mapping indicated that following reconsolidation of strong fear memory, the number of neurons activated in dorsolateral (LaDL) and ventrolateral (LaVL) parts of lateral amygdala increased compared to reconsolidation of weak fear memory. In addition, strong fear conditioning resulted in unique activation of several micro-regions in LaDL and LaVL indicating the topographical distribution of memory strength. We identified highly localized “hotspots” or regions of interests (ROIs) comprising a higher number of activated neurons with strong fear conditioning than with weak fear conditioning. These findings shed light on the spatial distribution of a fear memory in lateral amygdala during weak and strong fear conditioning. Administration of mirdametinib alone in mice with strong fear memory did not significantly reduce the number of pERK positive neurons whereas combined administration of DCS and mirdametinib significantly reduced the number of pERK-positive neurons in LaDL and LaVL. Importantly, topographic analysis suggested that study drugs reduced the number of pERK neurons in regions (ROIs) with higher activation in mice with strong fear memory. Importantly, these ROIs suggest specific and consistent ensembles of neurons in the LaDL and LaVL that are consistently activated by memory recall and are directly targeted by mirdametinib (Fig. 7).

At a molecular level, activation of NMDA receptors in glutamatergic neurons in LA results in the phosphorylation of extracellular signal-related kinase (ERK/MAPK) [27]. While the earlier research has shown that increase in pERK-positive neurons following consolidation and reconsolidation [18], the present study has shown for the first time that pharmacological inhibition of memory reconsolidation is associated with reduction in pERK-positive neurons in distinct subregions of LA. Activated ERK causes phosphorylation of CREB which is increased in the amygdala following reactivation of a fear memory [75, 76]. CREB activation leads to increased expression of immediate early genes (IEGs), *Arc* and *c-Fos* and further gene transcription and protein synthesis necessary for reconsolidation [14, 77, 78]. The above evidence suggests that inhibition of ERK/MAPK activation by drugs such as mirdametinib could be a useful therapeutic strategy to disrupt dysfunctional fear memories mediated by amygdala. Finally, as PTSD is associated with hyperreactivity of the amygdala [79], it could be postulated that mirdametinib might benefit PTSD patients by reducing the neuronal activation in amygdala and disruption of maladaptive fear memories.

The data from the present study suggest that memory strength could be governed by neurobiological underpinnings such as increased synaptic plasticity which may not reflect in freezing levels. Results from this study show there is a trend for the 3 × CU—US group to be slightly higher on both Day 2 reactivation and Day 3 memory text. However, as expected from the literature, changes in memory strength are not directly reflected as a change in freezing levels but rather as a change in susceptibility to disruption. In a study by Wang et.al., freezing levels after 1 CS-US pairing were similar to 10 CS-US pairings [80]. However, Wang and colleagues found the 10-pairing group was more resistant to extinction suggesting increased memory strength. Furthermore, the 10 pairing group was less susceptible to reconsolidation blockade by anisomycin compared to 1 pairing group.

An important consideration with the use of pharmacological targeting of memories, including using MEK inhibitors, is patient safety. Mirdametinib has completed Phase 2 clinical trial stage for treatment of cancer. Although mirdametinib at high dose (15 mg) was associated with side effects such as rash, diarrhea, peripheral edema, fatigue, dermatitis and retinopathy [33], it was effective and well-tolerated at the dose of 4 mg [33]. In toxicological studies in rodents, mirdametinib was safe at 10 mg/kg i.v., produced only minimal side effects (soft, reduced feces) at 30 mg/kg and severe side effects (hypoactivity, red staining of the muzzle, and soft/reduced feces) only at 100 mg/kg [81]. In the present study, only a single administration of mirdametinib at dose of 5, 10, or 25 mg/kg disrupted fear memory reconsolidation. This suggests that mirdametinib could be tested clinically at lower doses which are likely to be well tolerated. Moreover, the current study results suggest that mirdametinib does not affect memory processes in general as demonstrated by equal performance with vehicle on the Barnes maze test and has no effect on locomotor activity in open field test. This gives an indication that mirdametinib, at the reduced dose, provides a potential avenue for disrupting reconsolidation of fear memory clinically in PTSD patients, without producing adverse effects. However, this needs to be tested in clinical studies as the data regarding effects of mirdametinib on fear memory in humans is currently lacking.

To improve this research’s translational implications, future studies should include longer-term follow-ups to evaluate the persistence of memory. The present study was focused at memories that were recently consolidated in mice, whereas PTSD patients typically present to the clinic years after the traumatic event/s [82]. While mirdametinib was able to disrupt recent memories in this investigation, its application to older memories is yet unknown. The majority of research in this field focuses on the change of current memories, however, it is important to show the effectiveness of drugs including mirdametinib on old memories to more accurately represent the clinical presentation [82]. Review of reconsolidation studies suggests that recent memories are more susceptible to pharmacological intervention compared to old memories [69]. A possibility of interaction between different boundary conditions exists as strong memories which were resistant to disruption earlier but became susceptible when reactivation was done after a delay of 30–60 days [80]. In summary, mirdametinib’s effect on older memories will have to be evaluated before translation into clinical practice. Furthermore, while our study utilized sample size that was previously deemed sufficient to detect expected effects based on prior literature, increasing the number of mice per group could provide a more robust analysis. Nonetheless, the current findings provide novel insights into the role of clinically translatable MEK inhibitor and a solid foundation for future research.

**Fig. 8:**
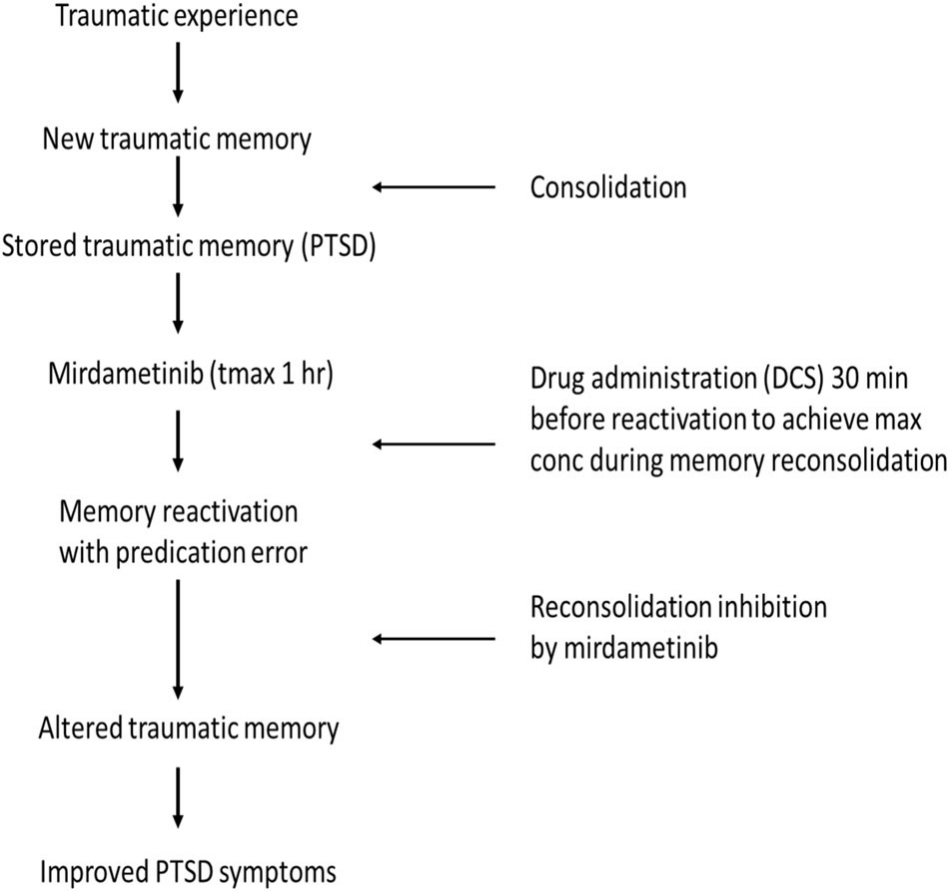
Proposed treatment protocol to disrupt trauma memory with mirdametinib to improve symptoms in PTSD patients.

**Fig. 9:**
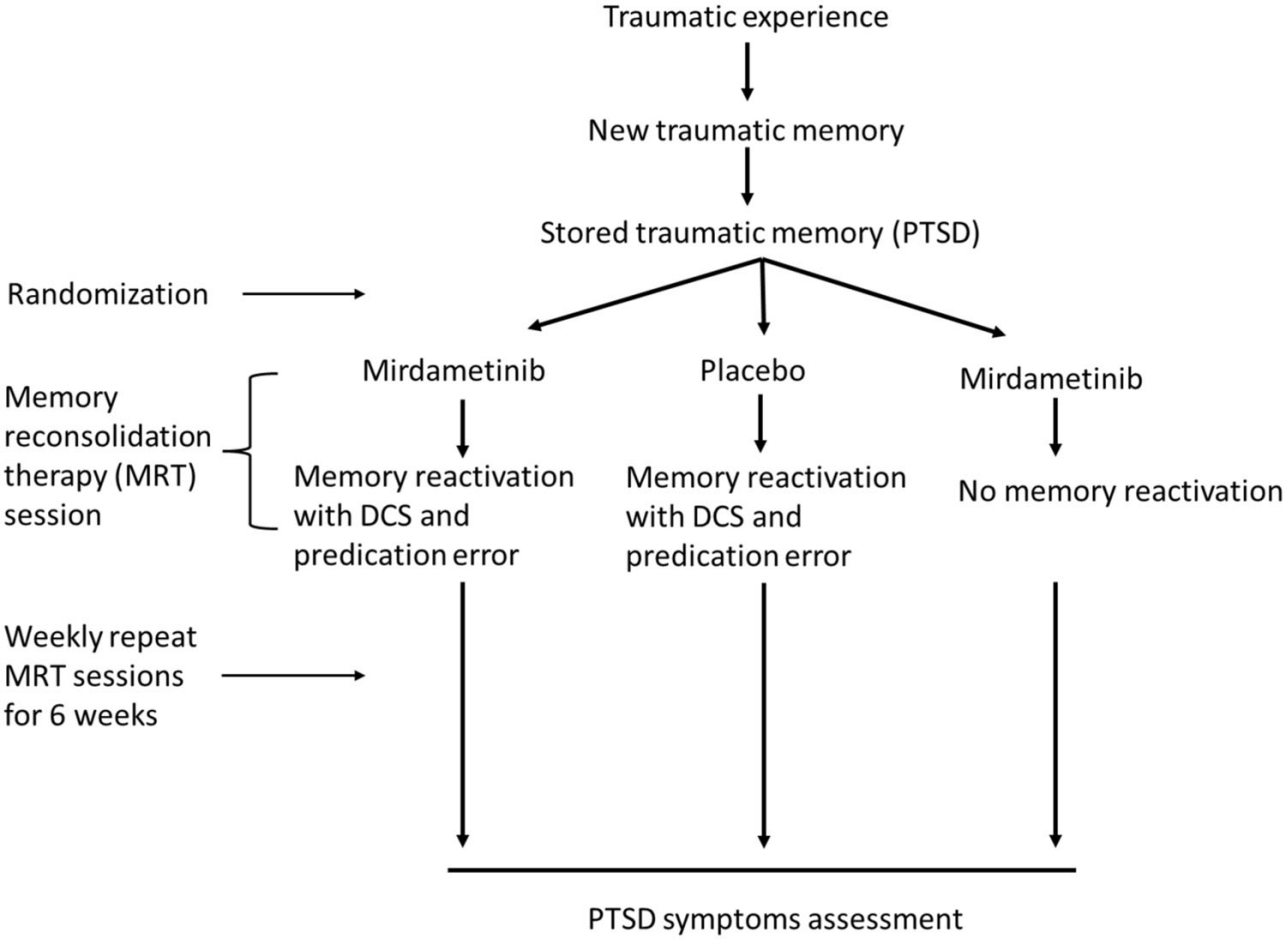
Proposed future clinical trial design to evaluate the effect of trauma memory disruption with mirdametinib on symptoms in PTSD patients.

The critical contribution of the present study is to show for the first time, the evidence for clinical translatable MEK inhibitor to disrupt weak as well as strong fear memories and further development of practical strategy for destabilization of strong fear memories that can be used in future clinical development of mirdametinib for PTSD. Future treatment plans for memory reconsolidation therapy in PTSD patients could incorporate DCS administration with mirdametinib to increase labilization of memory. For memory reconsolidation research to progress, it is important that memory destabilization methods be incorporated into clinical trial protocols. The present study emphasizes the advantage of combining mirdametinib with DCS to increase its efficacy in disruption of resistant memories. If mirdametinib is to be developed for memory disruption in PTSD patients, DCS administration prior to reactivation will be critical for its effectiveness. Mirdametinib is the first MEK inhibitor that can undergo clinical development for psychiatric indications such as PTSD. Results from the present study suggest that mirdametinib is a promising candidate for future clinical trials in PTSD patients. An improved treatment plan (Fig. 8) and clinical trial design (Fig. 9) with incorporation of memory destabilization strategy with mirdametinib administration in PTSD patients is proposed for future. During the initial visit a trauma memory script can be prepared followed by small but noticeable changes in script after administration of DCS and mirdametinib during follow up sessions to introduce prediction error and increase labilization of memory. This will improve the likelihood of translation of mirdametinib in clinical practice which has been lacking with other memory reconsolidation interventions tested so far. Similarly, future clinical trials could incorporate memory destabilization strategies (Fig. 9) to increase chances of success. Finally, mirdametinib has already been shown to modulate reward memories in the conditioned place preference model of addiction [35]. Thus, further exploration and clinical development of mirdametinib may offer new treatment avenues for not only patients with maladaptive fear memories like PTSD but also other psychiatric disorders.

## Conclusion

This study provides the first evidence that the clinically relevant MEK inhibitor, mirdametinib, when coupled with memory destabilization strategies including prediction error and DCS disrupts stronger fear memories. Moreover, when sequenced with memory recall, this effect is specific to fear memory and does not extend to other memory processes. Thus, mirdametinib can be developed clinically as a new drug for the treatment of PTSD.

## Supplementary information

Supplementary Material

## Data Availability

All relevant analyses for this paper are described in the manuscript. Data supporting the findings are available from the authors upon reasonable request.
