# Supplementary material for: The clinically relevant MEK inhibitor mirdametinib combined with D-cycloserine and prediction error disrupts fear memory in PTSD models
Source: Transl Psychiatry. 2024 Dec 18;14:492. doi: 10.1038/s41398-024-03190-6 (PMC11655561; doi:10.1038/s41398-024-03190-6)
Supplement: Supplementary file 1 — Supplementary Material [file 41398_2024_3190_MOESM1_ESM.docx]

Figure S1: Pre-CS data was comparable between groups in Experiment 1. Analysis with one-way ANOVA did not show significant differences between groups on Day 2 (Reactivation; p = 0.31) and Day 3 (Test; p = 0.26).

Figure S2: Pre-CS data was comparable between groups in Experiment 2. Analysis with one-way ANOVA did not show significant difference between groups on Day 2 (Reactivation; p = 0.34) and Day 3 (Test; p = 0.79).

Figure S3: Pre-CS data was comparable between groups in Experiment 3. Analysis with one-way ANOVA did not show significant difference between groups on Day 2 (Reactivation; p = 0.71) and Day 3 (Test; p = 0.69).

Figure S4: Pre-CS data was comparable between groups in Experiment 4. Analysis with unpaired T-test did not show a significant difference between groups on Day 2 (Reactivation; p = 0.84) and Day 3 (Test; p = 0.35).

Figure S5: Pre-CS data was comparable between groups in Experiment 6. Analysis with one-way ANOVA did not show significant differences between groups on Day 2 (Reactivation; p = 0.98) and Day 3 (Test; p = 0.97).

Figure S6: A single dose of trametinib (5 mg/kg) given after the recall without predication error failed to disrupt fear memory. Unpaired T test did not show significant difference between the groups on Day 2 (Reactivation; p = 0.40) and Day 3 (Test; p = 0.63).
